# Classification of video sequences into chosen generalized use classes of target size and lighting level

**DOI:** 10.1007/s11042-013-1546-1

**Published:** 2013-06-20

**Authors:** Mikołaj Leszczuk, Łukasz Dudek, Marcin Witkowski

**Affiliations:** AGH University of Science and Technology, al. Mickiewicza 30, 30059 Kraków, Poland

**Keywords:** Subjective evaluation techniques, Objective evaluation techniques, Quality of experience, Implementation

## Abstract

The VQiPS (Video Quality in Public Safety) Working Group, supported by the U.S. Department of Homeland Security, has been developing a user guide for public safety video applications. According to VQiPS, five parameters have particular importance influencing the ability to achieve a recognition task. They are: usage time-frame, discrimination level, target size, lighting level, and level of motion. These parameters form what are referred to as Generalized Use Classes (GUCs). The aim of our research was to develop algorithms that would automatically assist classification of input sequences into one of the GUCs. Target size and lighting level parameters were approached. The experiment described reveals the experts′ ambiguity and hesitation during the manual target size determination process. However, the automatic methods developed for target size classification make it possible to determine GUC parameters with 70 % compliance to the end-users’ opinion. Lighting levels of the entire sequence can be classified with an efficiency reaching 93 %. To make the algorithms available for use, a test application has been developed. It is able to process video files and display classification results, the user interface being very simple and requiring only minimal user interaction.

## Introduction

The transmission and analysis of video is frequently used for a variety of applications outside the entertainment sector, and is generally used to perform specific tasks. Examples of such applications include security, public safety, remote command and control, tele-medicine [1], and sign language. The Quality of Experience (QoE) concept for video content used for entertainment differs significantly from the QoE of video used for recognition tasks, because in the latter case the subjective satisfaction of the user depends on achieving the given task, e.g. event detection or object recognition. Additionally, the quality of video used by a human observer is distinct from objective video quality used in computer processing [5, 7]. This article is divided as follows. Section 2 describes the general approach to video classification adopted in the paper. Section 3 explains how the concept relates to user experience, as has been determined through an experiment with human subjects. Section 4 gives more detail about the methodology of the original experiment. The testing of the method’s accuracy and results obtained are discussed in Section 5. Section 6 includes the description of a test application which implements the algorithms developed and a user interface. Section 7 mentions some issues on which future efforts should concentrate. Section 8 sums up the paper.

## A framework for describing public safety video applications

The VQiPS (Video Quality in Public Safety) Working Group, established in 2009 and supported by the U.S. Department of Homeland Security’s Office for Interoperability and Compatibility, has been developing a user guide for public safety video applications. The aim of the guide is to provide the potential users of public safety videos with links to research and specifications that best fit their particular application. The process of developing the guide will have a further beneficial effect of identifying areas in which adequate research has not yet been conducted, so that such gaps may be filled. A challenge for this particular work is ensuring that it is understandable to users within public safety, who may have little knowledge of video technology.

The approach taken by VQiPS is to remain application-independent. Instead of attempting to individually address each of the many public safety video applications, the guide is based on their common features. Most importantly, as mentioned above, each application consists of some type of recognition task. The ability to achieve a recognition task is influenced by many parameters, and five of them have been selected as being of particular importance. They are:

- **Usage time-frame.** Specifies whether the video will need to be analysed in real-time or recorded for later analysis.
- **Discrimination level.** Specifies the level of detail required from the video.
- **Target size.** Specifies whether the anticipated region of interest in the video occupies a relatively small or large percentage of the frame.
- **Lighting level.** Specifies the anticipated lighting level of the scene.
- **Level of motion.** Specifies the anticipated level of motion in the scene.

**Fig. 1:**
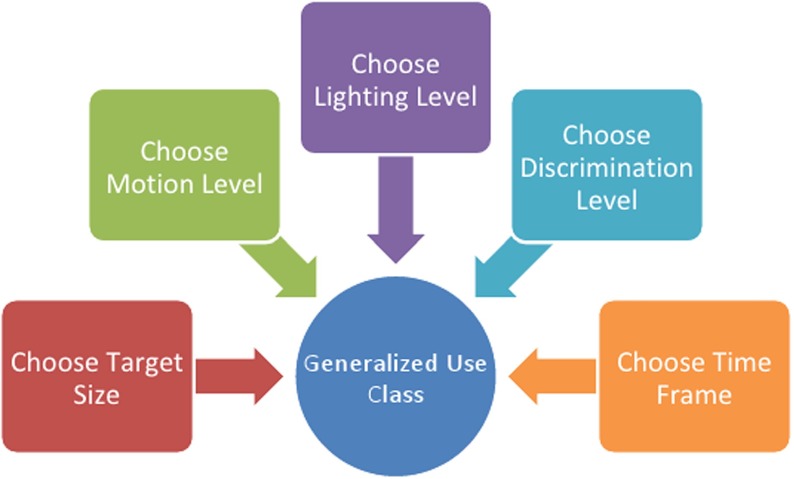
The structural diagram of generalized use classes video classification as proposed by VQiPS

These parameters form what are referred to as Generalized Use Classes, or GUCs [12]. Figure 1 is a representation of the GUC structural diagram.

## Classification of video sequences into specified generalized use classes

The aim of our research was to develop algorithms that would automatically assist classification of input sequences into one of the GUCs. Usage time-frame and discrimination level are scenario-related parameters and they cannot be set solely based on video analysis. As researchers cooperating with VQiPS, had previously conducted some research on level of motion, we approached the remaining parameters: target size and lighting level. A description of the GUC aspect does not define particular characteristics of the targets. Therefore, the description could not be used as a source of criteria for automatic algorithms. The first task was to obtain opinions of end-users, such as police officers, about essential objects included in the footage obtained from video surveillance and their size and lighting level. It was necessary to create an intuitive and easily accessible tool that would perform the research. A web-based interface was created, allowing end-users to perform the following tasks:

- watch the video sample,
- select targets and describe them,
- select the lighting level of the whole sequence and particular targets.

**Fig. 2:**
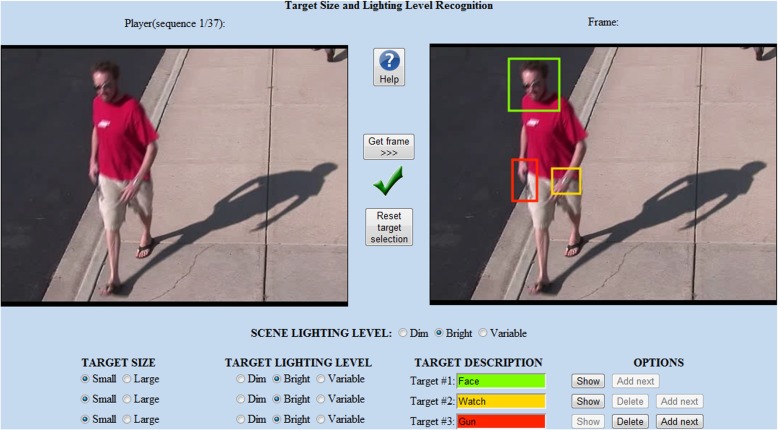
The interface

Figure 2 presents the developed tool.

Since the experiment was not described before (according to [2, 3], no formal rules do exist), the assessment of the testing tool (number and set of sequences, performance and outlook) was conducted on the basis of our opinion. Each test consisted of 37 sequences: 19 unique ones, and 9 pairs of the original size and the zoomed-in perspective. In line with the assumptions, the interface allowed users to select any target (up to 10 for 1 sequence) and specify its size and level of illumination for the entire sequence. The set of answers consisted of 616 target selections.

Due to the subjective nature of the experiment, manual validation was performed to prepare data for further analysis and reduce its ambiguity level. The excluded entries contained:

- two or more targets selected at once,
- no particular target selected,
- the same target selected more than once by one of the end-users.

After the validation process, there were 553 reasonable answers.

We have observed some answer instability with respect to particular targets, but not with respect to particular subjects (end-users). Actually, the general methodology for irrelevant subjects removal for recognition tasks has been not fully established yet [4, 6].

The process of analysing the answers – *tp* (true positive), *fp* (false positive), *fn* (false negative), and *tn* (true negative) – obtained from end-users for target size and lighting level, is based on measuring the $Precision=\frac{tp}{tp+fp}$, $Recall=\frac{tp}{tp+fn}$, $Accuracy=\frac{tp+tn}{tp+tn+fp+fn}$ and $F_1=2\cdot\frac{Precision\cdot Recall}{Precision+Recall}$ 1 scores. The process is described below.

### Target size

The user guide for public safety defines two sizes of the anticipated region of interest—small and large. Since there are only two levels of the target size, the results obtained from end-users were analysed with the aim of finding a criterion for binary classification. The first stage of the analysis was the rejection of inconsistent and ambiguous responses. A unique methodology of validating and grouping users’ answers was conducted in order to prepare the results for further computing. Next, different numerical representations of target sizes were calculated. Based on the F1 score, measuring accuracy, precision and recall, the best result was obtained using a size metric defined as the ratio of the largest size of the selected target to the appropriate length of the frame dimension.

**Fig. 3:**
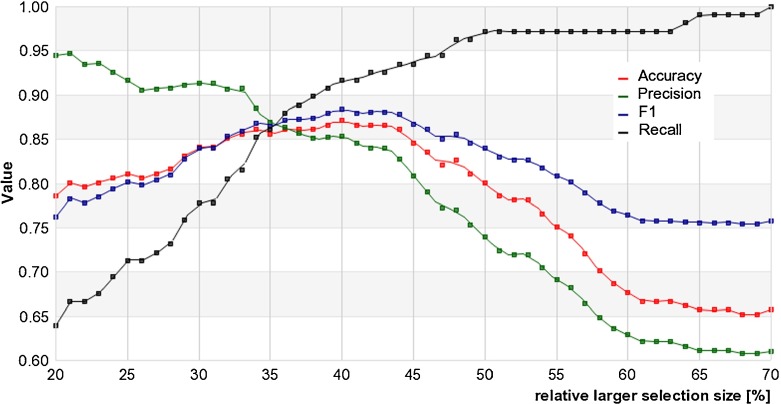
Statistical measures of the target size classifier as a function of the size metrics

Figure 3 presents the values of statistical tests for different metric values. As the graph shows, the maximum accuracy and F1 score values were obtained for a size metric equal to 40 % of frame size.

### Lighting level

**Fig. 4:**
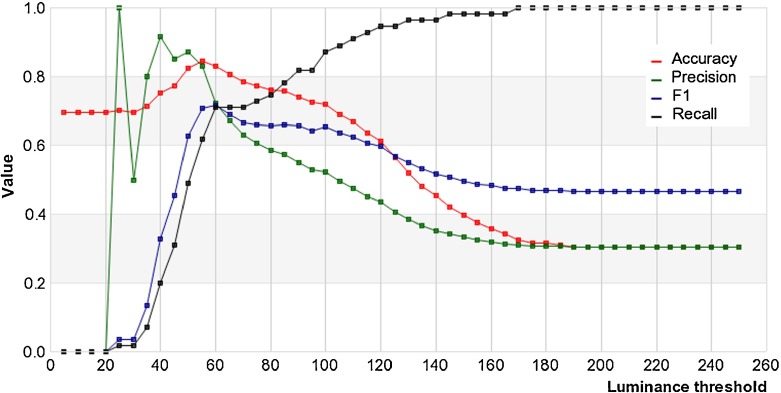
Statistical measures of the lighting level classifier as a function of the luminance threshold

The illumination of a video sequence can be described as dim, bright or variable. According to GUC descriptions, the lighting level refers to the entire sequence; however, in order to increase the stability of the results in the process of finding a classification method, the responses selected for each target were also taken into account. In addition, the low stability (ambiguity of collected responses) of “variable” responses implied their rejection. After that, we proceeded with the classification into two levels (dim and bright). Different methods were used to determine the numerical values representing illumination levels. The best results were obtained when calculating the average luminance of the selected target. Figure 4 presents the statistical measures of the lighting level classifier for different values of a luminance threshold (as expressed in [0,255] pixel units). The highest accuracy occurs for luminance equal to 55.

## Methods for automatic classification of entire generalized use class sequences

**Fig. 5:**
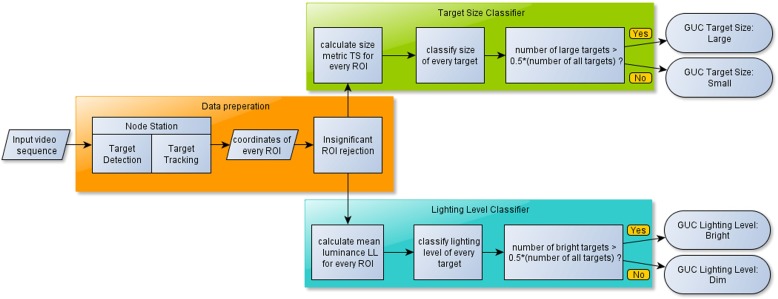
Block diagram of specified GUC classification method, further explanations were provided in the succeeding subsections

After determining the thresholds for the binary classifiers of GUC lighting level and target size, the work focused on automatic methods for classifying sequences. Because our study was not devoted to research into target detection and tracking, we used data generated by the appropriate methods implemented using third party software (Node Station) [8]. Analysis and further work on algorithms was performed in Mathworks Matlab [11], based on XML files generated by the Node Station during analysis of the input sequences. They contained coordinates of every detected and tracked target within the frames. Saving the data into XML files provided flexibility of further usage. As a result, the algorithms we worked on were based on image processing of each video frame. The algorithms were segmented into two steps: preparation of data obtained from the Node Station into a set of significant region of interest (ROI) coordinates, and determination of the size and lighting level for each target on the basis of pre-defined metrics. To evaluate automatic classifiers, sequences used in a previous study on thresholds during web tests performed by end-users, were used as input files. These footages were manually divided into two equal sets: testing and training. Figure 5 presents the developed classification method.

### Data preparation

The first module of the method needs to detect, track and numerically describe targets within the existing input sequence. This research was not restricted to the development of these methods, therefore we applied qualitative checks to the performance of the tools available to us, which were scripts supported by the Computer Vision System Toolbox provided with Matlab R2011b, and the Node Station detection and tracking system. The performance of the first was poor, and it would require further development in order to achieve efficiency similar to the second. As a result, the Node Station software was used in this research.

The object detection module implemented in the Node Station is based on a Gaussian Mixture Models of a background and the codebook method [8]. In this research we used the first detector mode, which means that objects were detected on the basis of picture elements that differ from the background [10, 9]. The aim of the Node Station authors was to use the system to detect moving objects within a static area. For the sequence in which the background was moving (for example footage taken in a car during a pursuit), the system detects multiple objects, supposedly in a random manner. Therefore, it was necessary to impose similar assumption to the input footage of our system. The set of sequences that did not meet these conditions was rejected. In order to use this set, it would be necessary to process files with other detection and tracking systems that would recognize objects in the footage captured with a moving camera and detect static objects.

**Fig. 6:**
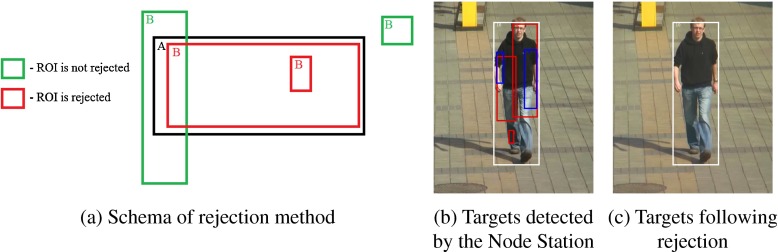
Reduction of detected redundant objects

One of the features of the detector was that frequently the same object was detected several times for one frame. As a result, any further calculations would give incorrect results. Therefore a module that filters the unwanted selections so that the remaining ones point out objects in an unambiguous manner was implemented. The rejection of redundant selections is presented in Fig. 6. When comparing rectangles A and B, the script removed rectangle B if its centre was within rectangle A and its height and the width were not more than 110 % of the height and the width of rectangle A. This value was obtained empirically, by comparison of the rejected selections at sequences used in the research. The goal was to generate a method that would reject multiple selections of targets whose dimensions were frequently only slightly larger, and leave the ones significantly larger.

The purpose of the above modules was to prepare the data in such a way as to enable further classifiers to analyse the error-free selection sets which contain all possible objects for each frame. It was also important not to lose the data which binds selections of successive frames into groups that may be analysed as a single target afterwards.

### Target size classifier

Based on the results of the experiment described in Section 3, a size metric equal to 40 % was used as a threshold in a binary classifier of target size. The first task for the classifier is to determine the size metric for every significant region of interest. It is calculated by dividing the larger side of the target selection by respective frame dimension as follows:

1 $$ TS = \frac{{\rm max}(x,y)}{{X}\veebar{Y}}\label{eqn:TS} $$

where *TS*—Target Size metric, *x*,*y*—size of selected ROI, and ${X}\veebar{Y}$—respective length of frame dimension.

Based on the TS, every selection is classified as large if *TS* > 40 %, and as small in all other cases. The size of each target is obtained by a majority of sizes of the selection of the same target during the entire sequence. After that, the GUC target size parameter is determined as large if number of large targets (not selections) is greater or equal to number of small targets, and small otherwise.

### Lighting level classifier

The lighting level is selected by comparing the average luminance with the value of 55—the threshold for which the highest accuracy occurs, as mentioned in Section 3. Classification starts with calculating of the mean luminance for every region of interest obtained in the data preparation step. Firstly, the entire selection is converted into grey scale, and the mean luminance is calculated. This value is compared to the value of 55 to determine lighting levels of each ROI. Based on data from the tracker, lighting levels of each target are the same as the majority of lighting levels of its selections. After that, the GUC lighting level parameter is determined by the majority of answers for all targets.

## Method evaluation

The evaluation process of the developed method was devoted to numerical counting of the number of algorithm outputs consistent with those obtained from end-users. Since their answers were not consistent, the ground-truth lighting level for the entire sequence was defined as the scene-lighting level, which was marked by two thirds of users. Since the detector we used was not able to detect certain objects, we had to make particular assumptions that determined which targets could be compared during the evaluation of our algorithm. One moving group of pixels was generally identified as a single object, although users recognized at least two. For example, in the sequence of a woman carrying a gun, software could not detect the face and the arms as separate objects. Therefore we decided that:

- groups of moving objects are selected as a single target (for example, a group of running people),
- parts of targets moving together cannot be detected (for example, the face of a robber),
- if two or more selection overlap, the larger one is taken into account.

The target size of the entire sequence was determined when two thirds of the targets were consistent with assumptions commonly made by end-users. Therefore, two sequences had to be rejected, since the number of same-size targets was below 66.67 % ($\frac{2}{3}$). In such situations it was assumed that since end-users cannot declare a particular target size, it also cannot be classified automatically by our system.

In the evaluation phase, footage that did not satisfy the detector assumptions (static background and moving objects) was rejected. In order to use footage that has been removed, a different detection system is necessary. The remaining sequences were randomly divided into testing and training sets. They consisted of ten sequences each for the size classifier and eleven each for the lighting level. Despite the small number of sequences, the development of classification algorithms allows us to reach a correlation with end-users opinions of 70 % for object size, and 93 % for the level of illumination. The low accuracy value of the target size classifier can be regarded as a weak result; nevertheless the method was developed and evaluated on the basis of inconsistent evaluation by end-users. In Fig. 3, accuracy values and F1 scores for the size metric ranging between 30 to 45 % are relatively close to the maximum. It implies that a binary classification of object size is not a trivial task for experts. The public safety guide proposed by VQiPS defines just two levels of target size, therefore it can be assumed that this classification also imposes an error margin on the algorithm itself.

## Implementation

The application runs on Windows. It was written in C+ +, using the functionality provided by the OpenCV library. The computations consist of two main stages. The first involves the analysis of consecutive frames in search of moving objects, and following their tracks in time. During this stage data involving object motion and luminance values is gathered. It is organized as sequences storing relevant parameters on a per object basis. In the next stage, the previously collected information is used to obtain individual classifications for objects, namely the size and brightness classes. The operators used to convert number sequences to binary categories are described in [13]. These partial results are then combined and the outcome is a general category applying to the whole scene.

### Blob detection

The first thing to do is to detect groups of pixels which change their position relative to neighbouring areas. There are many ways to achieve this; however, as the project only continued as far as the prototype stage, the solution adopted was a simple one. It relies on frame differences to locate the points where the most significant changes occur. For this reason, the method applies poorly to video sequences with a lot of changes in lighting and noise, and is not suitable when the background itself is not stationary. In any case, it should be noted that the standard approach based on background modeling will also fail in the presence of significant camera movement or unexpected changes of illumination. A frame is read from video stream, the previous one is subtracted from it, resulting in a difference image. Its absolute value is converted to greyscale and changed to a binary image by comparing it with a threshold, whose value is set to about 0.03 of the maximum luminance possible for a given numeric type. This value was chosen experimentally with the videos used in the original paper. It was set so as to filter out most of noise, compression artifacts etc. The resulting binary image undergoes morphological processing. To remove false positives resulting from noise and minor displacements in the background, morphological opening is performed. Points incorrectly omitted from the foreground may be filled and continuity of shapes restored using closing. After binary image pre-processing, contiguous regions of foreground must be detected and assigned distinct labels. This can be achieved straighforwardly in Matlab, but the functionality is not supported by OpenCV. Hence another library had to be involved in the process. As a solution, the CvBlob project was chosen. It enables clustering pixels into blobs; that is, finding continuous groups of points. When these are determined, some statistical data can be extracted for each one. This includes the centroid position, area, average luminance, pixel lists and some other variables useful for further operations. Each pixel group found should represent a moving object. In practice this is not always the case. After the previous operations, there may still be some areas where the high value for temporal difference is just a result of lighting variations or compression artefacts. They can mostly be dealt with by considering their size, as they are typically small compared to properly identified objects. After the blob detection region areas have been analysed, those which do not exceed a specified limit are rejected as false positives. In this case the threshold was set to 0.005 of the frame area, but this value is not meant to be decisive, and does not seem to require fine-tuning. In many cases, the resulting set of blobs is still not a sufficiently accurate representation of areas in motion. Differential analysis obviously tends to prefer regions at the edges of objects, where changes between frames are most evident. For homogenous parts of then moving foreground, it may result in producing hollow contours, which morphological operations cannot successfully fill. For the same reason, the objects sometimes become split into more parts. Additionally, when a region in motion slows down and rests in one place, it will not show up in the differential image. These issues are dealt with in the succeeding steps.

### Track following and recording data

Blobs are found in every frame of video. The concept of GUC requires the extraction of individual objects existing throughout a number of frames, and following their location to obtain a data series spanning the whole life of an object. Pixel groups detected each time must be correctly identified as snapshots of spatiotemporal tracks which exist for a longer period. The track maintaining module follows the positions of objects and allows for rejecting short-lived mistaken detections. Its operation is simple and also provided by CvBlob. Just like many parts of the program, it was intended to be replaced with a more sophisticated approach. In any case, it works relatively effectively in most cases, and reduces the influence of some temporary errors in blob determination and makes it possible to keep track of an object when it remains still over a period of time, or if only a part of it moves. The solution implemented in CvBlob uses a proximity matrix, which contains distances between pairs of blobs found in consecutive frames. It is assumed that corresponding ones should lie close to each other, as velocities are limited. This way, regions can be matched and motion history can be recorded. The algorithm is not robust and its capabilities are limited, but it proved sufficient for the majority of test scenes considered. Its operation depends on some preset parameters which influence the time scale of reactions to losing objects from sight or new ones being detected. Choosing correct values makes it possible to keep the information about tracks which are temporarily lost due to low level of motion and rejecting mistaken detections that do not survive long enough to be considered correct. Every time the algorithm tries to match detected blobs to existing tracks. When a blob does not fit, a new track is created for it. Necessary information, like width and height etc. is inserted into appropriate vectors. If a track is not matched to any blob for a specified number of times, it is not continued.

### Data series processing

When the whole scene is analysed by the tracker, each track record is available for further steps in the computation process. A dataset for an object consists of the following: both centroid coordinates, width and height of its bounding box, area, and average luminance. Each of the above is a series with values for every frame. The object’s lifetime is not necessarily the full duration of the video, so values from some points in time may be missing. In such case, a NaN value is present which indicates that a point in time must be omitted from the calculation. Every object is classified according to the data referring to it. The classification includes the size class, which can be small or large, and the brightness class, which is low or high. If the track record contains too few values other than NaN, or in other words has existed for too short a period of time, it is discarded as a result of errors in foreground detection. For size, the following procedure is applied. For each index in sequence where the object state is valid, the larger side of bounding rectangle is divided by the frame dimension parallel to it. If the result is greater than 0.4, the object is considered large for this particular moment. The size class for the whole track is the one which persists throughout the majority of its lifetime. The brightness classification for an object is calculated similarly. Series elements representing luminance values are compared with the previously determined threshold set to 55 (out of 255 pixel units). The object is considered bright if the luminance is over the threshold for the majority of its lifetime.

The classification for the whole scene is determined using the results for individual objects. The procedure again relies on the majority of binary indications. The target size category for the video is determined as the one that applies to more objects. The same happens with brightness. If no moving objects are detected, the categories are left undefined. Another issue is the absence of motion level estimation, which does not have a meaningful definition, and human research has not been conducted. In this situation binary categories cannot be assigned, and the result is always set to undefined.

### User interface

**Fig. 7:**
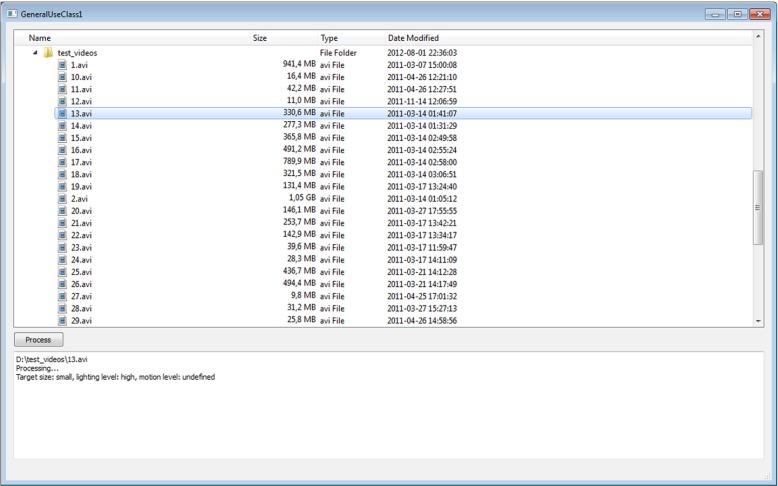
Reference application for practitioners, quantifying GUC—screen-shot of user interface

The front-end part of the application was developed based on the Qt framework. The user interface is simple, because using the application is intended to be straightforward. Further, the concept of GUC is intended to require no additional input. The main window (Fig. 7) is split horizontally into two areas.

The upper one contains a file browser. The lower is for text output. The user chooses an item from a file system tree. Files which extensions indicate video data are available for selection, others are greyed out and inactive. Double clicking a file or clicking the Process button initiates computation. The chosen file is analysed, and after processing the result appears in the text field. For small input files, it is almost instantaneous, and if calculations require more time, a progress bar appears. The interface is blocked until the working thread operation is finished. When the progress bar is visible, processing can be cancelled any moment. If the calculations are allowed to reach the end, the classification for the scene appears in the text field.

## Further development

The main issue that emerged during the evaluation of automatic methods of classification into specified GUCs was the imperfection of the detector. It can be assumed that the development of this module by the implementation of the following methods would significantly improve the range of applications for the classification system:

- detection of sub-objects (such as a weapon),
- detection of stationary objects (such as abandoned luggage),
- detection of targets at sequences containing moving background (such as footage recorded in a car during pursuit).

Such methods are likely to reduce the number of wrongly detected targets, and make our algorithm more precise. Target size is also an important issue in motion analysis, therefore this research is also a contribution to a study on automatic classification of motion leads by the VQiPS into GUCs.

## Conclusion

The aim of our research was to develop algorithms that would automatically assist classification of input sequences into one of the GUCs. Usage time-frame and discrimination level are scenario-related parameters and they cannot be set solely based on video analysis. As researchers cooperating with VQiPS, had previously conducted some research on level of motion, we approached the remaining parameters: target size and lighting level. The results of the experiment revealed that the problem of binary classification of target size is non-trivial and difficult to determine by experts. Consequently, the developed automatic method of target size classification allows us to determine the GUC parameter with compliance levels of 70 % with end-users opinion, which is a satisfactory result indicating the indecision of users. Lighting levels of the entire sequence can be classified with an efficiency reaching 93 %. A user guide for public safety defines very common features of video sequences. As a result, the classification into GUCs of any footage by a computer algorithm cannot be taken as a certain result, therefore it should be verified manually.

## Footnotes

1 Also known as F-score or F-measure, used to measure the test accuracy.

## References

[1] Duplaga M, Leszczuk M, Papir Z, Przelaskowski A (2008). Compression evaluation of surgery video recordings retaining diagnostic credibility (compression evaluation of surgery video). Opto Electron Rev.

[2] International Telecommunication Union (1999) ITU-T P.910, subjective video quality assessment methods for multimedia applications. http://www.itu.int/rec/T-REC-P.910-200804-I. Accessed 11 June 2013

[3] International Telecommunication Union (2008) ITU-T P.912, subjective video quality assessment methods for recognition tasks. http://www.itu.int/rec/T-REC-P.912-200808-I. Accessed 11 June 2013

[4] Janowski L (2012) Task-based subject validation: reliability metrics. In: Fourth international workshop on quality of multimedia experience (QoMEX) 2012, pp 182–187. doi:10.1109/QoMEX.2012.6263863

[5] Janowski L, Kozłowski P, Baran R, Romaniak P, Głowacz A, Rusc T (2012) Quality assessment for a visual and automatic license plate recognition. Multimed Tools Appl. doi:10.1007/s11042-012-1199-5

[6] Leszczuk M, Janowski L, Romaniak P, Głowacz A, Mirek R (2011) Quality assessment for a licence plate recognition task based on a video streamed in limited networking conditions. In: Dziech A, Czyżewski A (eds) Multimedia communications, services and security. Communications in computer and information science, vol 149. Springer Berlin Heidelberg, pp 10–18. doi:10.1007/978-3-642-21512-4_2

[7] Leszczuk M, Stange I, Ford C (2011) Determining image quality requirements for recognition tasks in generalized public safety video applications: definitions, testing, standardization, and current trends. In: 2011 IEEE International symposium on broadband multimedia systems and broadcasting (BMSB), pp 1 –5. doi:10.1109/BMSB.2011.5954938

[8] Dalka P, Szwoch G, Ciarkowski A (2011) Distributed Framework for Visual Event Detection in Parking Lot Area, Multimedia Communications, Services and Security Communications in Computer and Information Science, vol 149, pp 37–45. doi:10.1007/978-3-642-21512-4_5

[9] Szwoch G, Dalka P (2008) Identification of regions of interest in video for a traffic monitoring system. In: Proc. 1st intern. conf. on information technology, pp 337–340

[10] Szwoch G, Dalka P, Czyżewski A (2008) Objects classification based on their physical sizes for detection of events in camera images. In: 2008 Signal processing algorithms, architectures, arrangements, and applications (SPA), pp 15–20

[11] The MathWorks, Inc. (2013) Matlab documentation. http://www.mathworks.com/help/techdoc/. Accessed 11 June 2013

[12] VQiPS (2011) Video quality tests for object recognition applications. http://www.safecomprogram.gov/library/Lists/Library/Attachments/231/Video_Quality_Tests_for_Object_Recognition_Applications.pdf. Accessed 12 June 2013

[13] Witkowski M, Leszczuk M (2012) Classification of video sequences into specified generalized use classes of target size and lighting level. In: IEEE International symposium on broadband multimedia systems and broadcasting, (BMSB) 2012, pp 1–5. doi:10.1109/BMSB.2012.6264239

